# Oral selective serotonin reuptake inhibitors activate vagus nerve dependent gut-brain signalling

**DOI:** 10.1038/s41598-019-50807-8

**Published:** 2019-10-03

**Authors:** Karen-Anne McVey Neufeld, John Bienenstock, Aadil Bharwani, Kevin Champagne-Jorgensen, YuKang Mao, Christine West, Yunpeng Liu, Michael G. Surette, Wolfgang Kunze, Paul Forsythe

**Affiliations:** 1 McMaster Brain-Body Institute at St Joseph’s Healthcare Hamilton, Hamilton, Canada; 2 Department of Pathology and Molecular Medicine, McMaster University, Hamilton, Canada; 3 Michael G. DeGroote School of Medicine, McMaster University, Hamilton, Canada; 4 Department of Medicine, McMaster University, Hamilton, Canada; 5 Farncombe Family Digestive Health Research Institute, McMaster University, Hamilton, Canada; 6 Department of Psychiatry and Behavioural Neurosciences, McMaster University, Hamilton, Canada; 7 Department of Biology, McMaster University, Hamilton, Canada; 8 Firestone Institute for Respiratory Health, St Joseph’s Healthcare Hamilton, Hamilton, Canada

**Keywords:** Excitability, Depression

## Abstract

The vagus nerve can transmit signals to the brain resulting in a reduction in depressive behavior as evidenced by the long-term beneficial effects of electrical stimulation of the vagus in patients with intractable depression. The vagus is the major neural connection between gut and brain, and we have previously shown that ingestion of beneficial bacteria modulates behaviour and brain neurochemistry via this pathway. Given the high levels of serotonin in the gut, we considered if gut-brain signaling, and specifically the vagal pathway, might contribute to the therapeutic effect of oral selective serotonin reuptake inhibitors (SSRI). Mesenteric nerve recordings were conducted in mice after treatment with SSRI to ascertain if this class of drugs resulted in increased vagal excitability. Patch clamp recordings of enteric neurons were carried out to measure activity of primary afferent neurons in the gut in response to SSRI and to assess the importance of gut epithelium in transducing signal. The tail suspension test (TST) was used following 14d feeding of SSRI in vagotomised and surgical sham mice to measure depressive-like behaviour. Brain mRNA expression was examined via PCR and the intestinal microbiome was assessed. Mesenteric nerve recordings in BALB/c mice demonstrated that oral treatment with SSRI leads to a significant increase in vagal activity. This effect was not observed in mice treated with a representative noradrenaline-dopamine reuptake inhibitor. It is known that signals from the gut can be transmitted to the vagus via the enteric nervous system. Exposure of the gut to SSRI increased the excitability of intrinsic primary afferent neurons in the myenteric plexus, through an intestinal epithelium dependent mechanism, and alpha-diversity of gut microbiota was altered. Critically, blocking vagal signaling from gut to brain, via subdiaphragmatic vagotomy, abolished the antidepressive effects of oral SSRI treatment as determined by the tail suspension test. This work suggests that vagus nerve dependent gut-brain signaling contributes to the effects of oral SSRI and further, highlights the potential for pharmacological approaches to treatment of mood disorders that focus on vagal stimulation and may not even require therapeutic agents to enter the circulation.

## Introduction

The vagus nerve is the tenth cranial nerve and is the main afferent pathway connecting the gut to the brain. Sensory information arrives from the gut via the nodose ganglia to the nucleus tractus solitarius in the brain, from which the afferent fibres ramify to widespread regions. Stimulating the vagus can lead to a significant reduction in anxiety and depressive-like behaviours in rats^1,2^, while clinically vagal stimulation is an FDA approved treatment for intractable depression and also has been used in the treatment of refractory epilepsy^3^.

In the past decade, we and many others have been involved in furthering the concept of a gut-brain axis focusing on the role of potentially beneficial commensal gut bacteria on behaviour and brain function^4–7^. We previously showed that oral treatment with a specific microbe, *Lactobacillus rhamnosus* JB-1, was able to mediate anxiolytic and antidepressive-like behaviour through a mechanism dependent on gut-brain signaling via the vagus nerve^8^. *Lactobacillus rhamnosus* JB-1 was shown to evoke vagal activity in mesenteric nerve afferents^9^, largely via modulation of intrinsic primary afferent neurons (IPANs) of the enteric nervous system (ENS)^10^. Thus we have identified a link between the effect of gut lumen derived signals on ENS activity and antidepressant effects mediated via the vagus nerve.

Throughout the years, extensive research efforts have attempted to unravel the mechanisms of antidepressant action of selective serotonin reuptake inhibitors (SSRI)^11^. In spite of the fact that at least 80% of serotonin synthesis in the body occurs in the gut^12^, it is commonly argued that SSRI act via their inhibitory action on the serotonin transporter in the brain, thus preventing presynaptic neuronal reuptake of serotonin. However the view that this is how SSRI exert their antidepressant activity is controversial and not universally accepted^13^, and little or no attention has been paid to the possibility that the therapeutic effects of SSRI may be the result of their primary actions in the gut and not the brain. Given the vast amount of serotonin in the gut and the fact that serotonin modulates ENS and vagal activity, we sought to examine the relationship between oral SSRI activity and vagal nerve signaling in mice, and to determine if subdiaphragmatic vagotomy might interfere with the central effects of oral SSRI in the tail suspension test (TST), a validated and well-recognized mouse behavioural test that measures rodent behaviour most consistent with depression in humans^14^. We show here that this is indeed the case, and that this effect is dependent on route of administration since vagotomy did not interfere with the antidepressant effects of parenteral injection. The dependency of oral SSRI on the integrity of the vagus nerve appears to be selective since its section did not prevent the antidepressant effect of an oral norepinephrine-dopamine reuptake inhibitor (NDRI). Our results may open the door to a novel view of the primary mechanisms of action of this class of orally active psychotropic drugs, and promote new approaches to influence behaviour via selective peripheral chemotherapeutic vagal stimulation.

## Methods

### Mice

Adult male BALB/c mice aged 6–8 weeks were obtained from Charles River (Montreal, QC, Canada) and allowed to habituate to the animal facility for at least 1 week. The mouse strain was selected based on the fact that it has an intrinsic anxiogenic-like phenotype^15^, is most consistently responsive to behavioural tests of despair^16^, our previous publication^8^, and our recent report that BALB/c but not Swiss Webster mice respond to SSRI treatment with antidepressive-like behaviour^17^. Mice were maintained on a 12 h light/dark cycle (lights on at 5 am) with *ad libitum* access to food and water.

### Vagotomy

Some animals were subjected to a subdiaphragmatic vagotomy with pyloroplasty as previously described^18^. Animals were allowed to recover for 7–10 days prior to 14d oral feeding with antidepressant treatments before behavioural testing and then harvesting the gut and brain for *ex vivo* experiments. Sham vagotomy was also performed on surgical control animals. We found no evidence of significant differences in weight gain 1 week postsurgery in either vagotomised or sham animals (see Additional File 1: Fig. S1A), a vital and reliable indicator of animal well-being post surgery. Indeed, of the 12 animals subjected to vagotomy, there were no fatalities or markers of distress. In addition, we found no evidence of vagal nerve regrowth when a subset of animals were tested following behaviour experiments via mesenteric nerve recording after CCK stimulation (see Additional File 1: Fig. S1B).

### Drug treatments

7–10 days postsurgery (or in age matched non-surgical controls) oral drug treatment delivery began. All oral delivery of antidepressants was via the drinking water. Animals randomized to the fluoxetine group received fluoxetine hydrochloride (Sigma Millipore) at a calculated individual dose of 18 mg/kg for 14 days^19^. Those in the oral sertraline group received sertraline hydrochloride (Sigma Millipore) at a dose of 6 mg/kg for 14 days, while those in the sertraline injected group received a single i.p. injection at a dose of 20 mg/kg 30 m prior to behavioural testing^19^. Mice in the bupropion group received bupropion hydrochloride (Sigma Millipore) at a dose of 6 mg/kg for 14 days^20^.

### Mesenteric nerve recording

Tissue was prepared from treatment naïve mice as described previously^21^. Briefly, 2 cm fresh segments of jejunum were placed in a 2 mL recording dish lined with Sylgard and filled with Krebs. Oral and anal ends were cannulated and flushed with plastic tubing and the mesentery was pinned out so that nerve bundles were isolated by microdissection. The serosal compartment was separately perfused with prewarmed Krebs/nicardipine (3 µM). The nerve bundle was gently sucked into a glass pipette with an attached electrode and extracellular multiunit nerve recordings were made using a Multi-Clamp 700B amplifier and Digidata 1440A signal converter (Molecular Devices). Baseline recordings were collected for 20 min with luminal perfusion of the Krebs prior to adding drug treatments at a concentration of 10 uM (equivalent doses to the respective oral treatments identified in methods). Electrical signals were bandpass-filtered at 2 kHz and sampled at 20 kHz. Single units representing discharge from individual single vagal fibres were discriminated and identified by their unique spike waveform shape and amplitude^22^ using Dataview computer software^23^, which uses principal component analysis to sort the recorded multiunit spikes into single unit categories according to shape and amplitude. Luminal perfusion experiments had an n = 54(5 mice) (fluoxetine), 58(5 mice) (sertraline), and 52(3 mice) (bupropion). Feeding experiments had an n = 112(10 mice) (Krebs), 30(5 mice) (fluoxetine), 69(8 mice) (sertraline) and 36(5 mice) (bupropion).

### Patch clamp experiments

Jejunal tissue was prepared for patch clamp of myenteric neurons using hemidissection as described previously^24^. Patch pipettes were pulled on a Flaming-Brown-P97 (Sutter Instruments, Novato, USA) electrode puller to produce 4–7 MΩ micropipettes. Signals were 4 point Bessel filtered at 2 or 5 kHz, then digitized to 5 or 20 kHz. Positive pressure was applied to the pipette as the tip entered solution until contact with the neuron was made. Whole cell recording mode began after suction and the amplifier was then switched to current-clamp mode. Recordings were filtered such that only those with resistances >4 GΩ were used. Epithelial stimulation and recordings were made as described previously^24^, using Multiclamp 700B Amplifier, Digidata 1440A signal converter and PClamp 10.7.0 software (Molecular Devices). Neuron excitability, AP, and membrane properties were assessed first with Krebs buffer in the epithelial compartment (~20 min) and then with inflow to the chamber switched to a solution containing the drug treatment. Patch clamp experiments had an n = 8/group.

### Tail suspension test (TST)

1 day following a 14d course of oral treatments, or 30 m following injected sertraline, animals were tested for depressive-like behaviour with the TST. Mice were transferred from the housing room to the behavioural testing room and allowed to habituate for 30 min. Following habituation, mice were suspended by the tail using 17 cm laboratory tape^25^ from a suspension bar. 2 cm of the tape was affixed to the mouse tail and the remainder of the tape used for suspension. Animals were suspended for a total of 6 min. Behaviour was video recorded and scored by a blinded observer. Freezing behaviour was measured and calculated as a percentage of the total time suspended. Following behavioural testing, mice were returned to the colony room and resumed oral treatment until sacrifice. All behavioural experiments had an n = 12/group.

### PCR

Following behavioural testing animals were killed, brains were removed and whole hippocampi were hand dissected and stored at 4 °C in RNA*later* RNA Stabilization Reagent (Qiagen) for 24 h, followed by storage at −80 °C until tissue processing. Total RNA was extracted using the *mir*Vana^TM^ total RNA extraction kit (Life Technologies) according to manufacturer’s protocol. RNA concentration and quality were determined using a Nanodrop 1000 (Thermo Scientific). RNA was reverse transcribed using high-capacity cDNA reverse transcription kit (Thermo Fisher Scientific) in a SimpliAmp Thermal Cycler (Applied Biosystems Catalogue #A24811). cDNA was used as a template for qPCR reaction using PowerUp SYBR Green Master Mix (Applied Biosystems, Life Technologies) containing ROX dye Passive Reference. qPCR reactions were performed using the QuanStudio3 machine (Applied Biosystems). Data were normalized to endogenous control GAPDH and the relative gene expression was analyzed using the ΔΔCt method. Genes of interest examined were brain-derived neurotrophic factor (BDNF), doublecortin (DCX), tropomysin receptor kinase B (trkB), and neuronal differentiation 1 (neuroD1). Primers sequences will be made available upon request. All PCR experiments had an n = 8/group.

### 16S rRNA microbiome analysis

Samples were collected and stored at −80 °C and DNA extraction was carried out as previously described^26^, with modifications to increase quantitative recovery of bacteria across taxa^27^. A modified, barcoded Illumina sequencing method^28^ was used for 16S rRNA gene sequencing. A MiSeq Illumina sequencer in the McMaster Genome Center was used to carry out paired-end reads of the V3 region (using 341F and 518R primers) and 250 nt paired-end sequencing. Data were processed through an in-house bioinformatics pipeline^29^, producing clustered sequences operational taxonomic units(s) using abundant OTU^30^, and taxonomic assignments using the RDP classifier^31^ and the Greengenes training set^32^. Sequencing produced 8367 OTUs, and a minimum, maximum, and median of 4675, 243597, and 52773 reads/sample respectively.

Using QIIME^33^, data were rarefied at the lowest sequencing depth for analysis, as previously described^26,34^. Simpson diversity index and Shannon diversity index were calculated for alpha-diversity analysis, and Jackknife resampling was used to generate Bray-Curtis distances for beta-diversity analysis.

### Statistical analysis

All statistics were performed with GraphPad Prism V7. Outliers were defined as more than 2 standard deviations from the group mean and removed in order to avoid type II error. Main effects and interactions were determined with ANOVA and Dunnett’s and Bonferroni’s post hoc analysis. Data from acute electrophysiology experiments were analyzed by paired t-test. Microbiome data was analyzed with permutational multivariate analysis of variance (PERMANOVA; 999 permutations), Mann-Whitney U tests, and DESeq. 2 (False Discovery Rates, q < 0.05)^35^. Two-tailed unpaired Student’s *t* test was used for weights. All significance thresholds were set at p < 0.05.

### Ethics approval and consent to participate

All experiments were conducted in accordance with the guidelines of the Canadian Council on Animal Care and were approved by McMaster University’s Animal Research Ethics Board, protocol number 16-12-42.

## Results

### Feeding SSRI but not NDRI stimulates increased vagal fibre activity in the mesenteric afferent nerve

**Figure 1:**
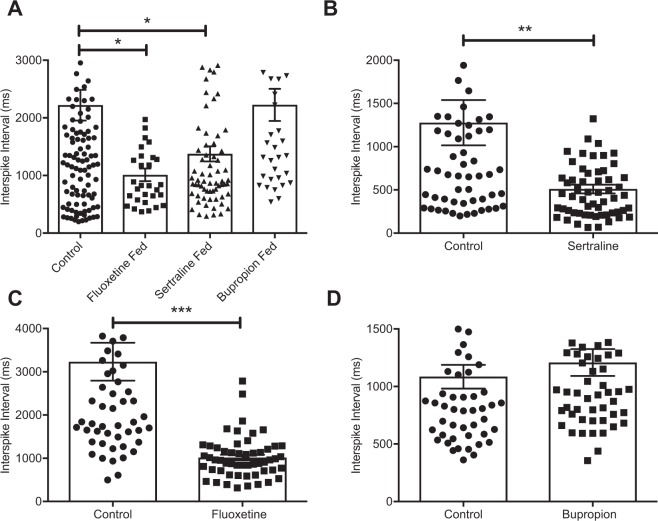
Orally delivered SSRI and acutely delivered SSRI to the intestinal lumen increase vagal firing. (**A**) Time interval between vagal spike firing following oral SSRI and NDRI feeding. (**B**) Time interval between vagal spike firing following acute presentation of sertraline to intestinal lumen. (**C**) Time interval between vagal spike firing following acute presentation of fluoxetine to intestinal lumen. (**D**) Time interval between vagal spike firing following acute presentation of bupropion to intestinal lumen. Statistics for acute experiments are paired, two-tailed t test and fed experiment one way ANOVA with Dunnett’s multiple comparison post hoc. Bars are means ± SEM, *P < 0.05, **P < 0.01, ***P < 0.001, (fed experiments, n = 5–8 animals/30–70 recordings/group, acute experiments, n = 5–10 animals/55–60 recordings/group).

In order to determine the effects of oral SSRI on the vagus, we recorded action potentials (AP) in vagal afferent fibres from gut jejunal segments removed from mice after ingestion of SSRI for 14 days^9^. Feeding with either sertraline or fluoxetine significantly decreased the mean interval between vagal spike firings (Fig. 1A), indicating an increase in firing frequency. Measuring firing frequency is a long established index of vagal fibre activity^36–38^. Feeding the NDRI bupropion for the same amount of time, did not change the interval between vagal spike firings as compared to water fed nonsurgical controls. This showed that bupropion was relatively inactive in the stimulation of vagal afferent fibres.

### Acute treatment with SSRI but not NDRI increases vagal fibre activity

We then wished to determine if acute luminal administration had the same effects on vagal firing as feeding, we thus perfused jejunal segments of naïve mice with SSRI. The addition of sertraline or fluoxetine directly to the gut lumen in separate preparations significantly reduced the interval between spike firings indicating increased vagal firing rates (Fig. 1B,C), while acute treatment with bupropion did not (Fig. 1D). Thus exposure of the gut to SSRI either via 14 days of feeding or directly and acutely, promoted an increase in the frequency of afferent vagal fibre firing in the mesenteric nerve bundle that carries gut derived signals to the brain. Feeding or direct exposure of the gut to bupropion had no such effect.

### SSRI increases excitability of enteric sensory neurons via gut epithelium

**Figure 2:**
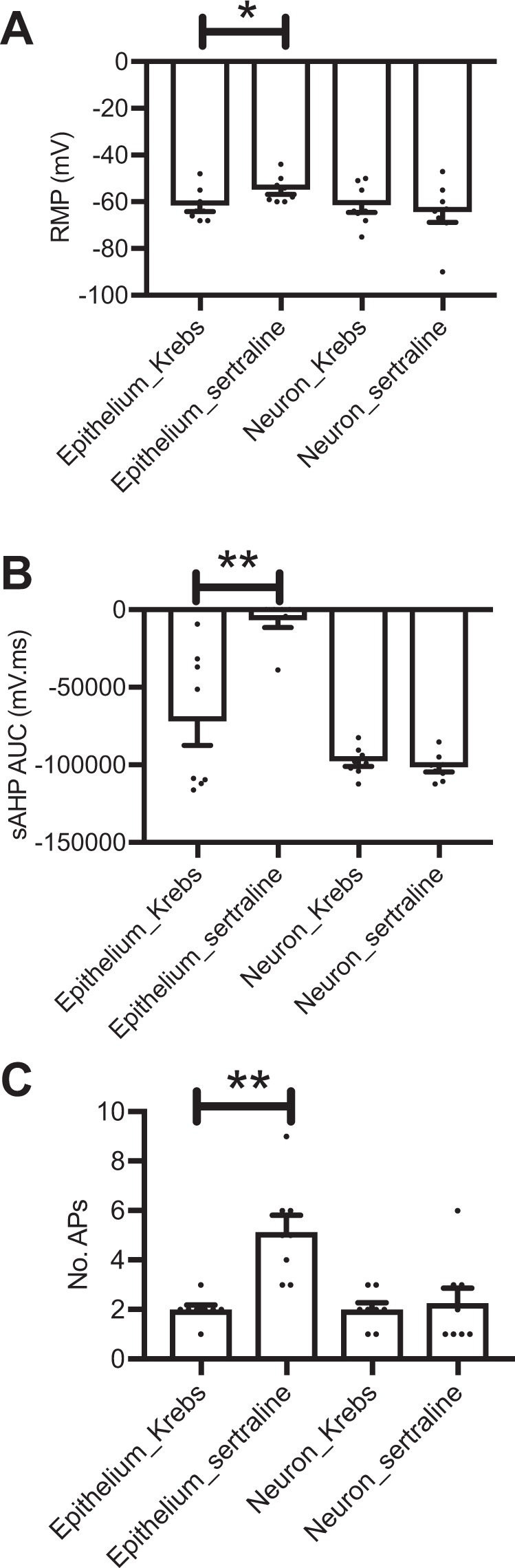
Sertraline added to the intestinal epithelium increases excitability of sensory enteric neurons. (**A**) Resting membrane potential of enteric sensory neurons after addition of sertraline vs. Krebs. (**B**) Duration of slow afterhyperpolarization of sensory neurons following addition of sertraline vs. Krebs. (**C**) Number of action potentials generated after addition of sertraline vs. Krebs. Bars are means ± SEM, *P < 0.05, paired two-tailed t test, **P < 0.01, (n = 8/group).

To obtain further information on the effects of the SSRI on IPANs of the myenteric plexus we employed a hemidissection model that we have previously described^24^. In this preparation neurons are exposed on only half of the area of an opened segment of jejunum and are separated by a vertical divider from the continuous full thickness mucosa. The neuronal exposed chamber has had the mucosa and circular muscle removed to allow myenteric plexus neurons to be directly patch clamped. Adding sertraline to the intact epithelium significantly decreased the resting membrane potential (RMP) of IPANs as compared to Krebs (Fig. 2A) however the addition of sertraline directly to exposed neurons did not alter RMP. Similarly, adding sertraline to the mucosal epithelium, but not Krebs, significantly reduced the slow afterhyperpolarization (sAHP) (relative refractory period) in the IPANs. Again, the addition of sertraline directly to exposed neurons had no effect on the sAHP (Fig. 2B). The number of AP generated in response to intracellular injection of depolarizing current at twice threshold intensity significantly increased after sertraline was added to the mucosal epithelium as compared to Krebs, but did not change when added directly to the exposed neurons (Fig. 2C). No differences were observed in either threshold required for AP or in leak conductance (data not shown). We conclude from these experiments that oral SSRI require an intact gut epithelium to effectively signal the vagus via the IPANs in the ENS, albeit through as yet unknown mechanisms.

### Oral SSRIs depend on an intact vagus for behavioural effect in mice

**Figure 3:**
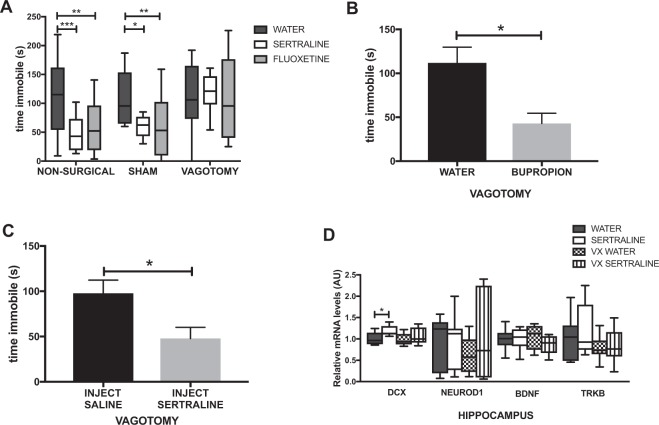
Orally delivered SSRI require an intact vagus in order to exert antidepressant effects. (**A**) Time immobile in the tail suspension test (TST) following oral SSRI treatment. (**B**) Time immobile in the TST following oral NDRI treatment. (**C**) Time immobile in the TST following parenteral sertraline treatment. (**D**) Relative mRNA gene expression of hippocampal DCX, NeuroD1, BDNF and TrkB following sertraline treatment in control and vagotomised mice. Bars are means ± SEM, *P < 0.05, **P < 0.01, ***P < 0.001, (behaviour, n = 12/group; PCR n = 8/group).

In a separate cohort of animals, we assessed the necessity for intact vagal signalling between gut and brain to mediate the effects of SSRI in the TST. We performed subdiaphragmatic vagotomy with pyloroplasty and sham surgery on male BALB/c mice^18^ and following recovery administered SSRI, (sertraline or fluoxetine, 6 & 18 mg/kg respectively), via drinking water for 14 days. On day 15, mice underwent TST to assess time spent immobile, an indirect method of inferring antidepressive-like behaviour in mice^14^. Not only did all vagotomised mice survive surgery and appear otherwise healthy post-recovery, vagotomy had no effect on animal weight after the recovery period, an important indicator of health status following surgery (Additional File 1: Fig. S1A). Animals in both non-surgical and sham surgery groups responded to both fluoxetine and sertraline with a reduced time spent immobile in the TST compared to water fed controls, indicating antidepressive-like behaviour for the treatment group. Animals in the vagotomy group did not display antidepressive-like behaviour in the TST following treatment with either SSRI (Fig. 3A). We then assessed the effects of an oral atypical antidepressant and NDRI, bupropion (6 mg/kg) which has no effect on serotonin synthesis or utilization^39^. Here we observed that vagotomy did not inhibit the antidepressant response in the TST (Fig. 3B). Finally we assessed vagotomised animals in the TST following an acute parenteral administration (20 mg/kg) of sertraline. We found that animals responded to the injected SSRI with antidepressive-like behaviour (Fig. 3C) indicating that while the primary effect of oral SSRI is dependent on the vagus, behavioural effects of these drugs are still observed after systemic administration irrespective of vagal integrity.

In order to ascertain that vagal nerve regrowth had not occurred by the time experiments were undertaken following surgical recovery and drug treatment, a separate cohort of mice was allowed to recover from vagotomy for 4 weeks and then subjected *ex vivo* to the same mesenteric nerve recordings as described, for responsivity to the selective vagal stimulant CCK. No increase in vagal nerve activity was observed following exposure, indicating the absence of afferent vagal nerve fibres (Additional File 1: Fig. S1B).

### Sertraline treatment upregulates some but not all markers of hippocampal neurogenesis in a vagal-dependent manner

Since SSRI have been posited to act in part by promotion of hippocampal neurogenesis^40^, brains were collected on day 18 and hippocampi dissected out for PCR analysis of mRNA gene expression of 4 biomarkers related to neural proliferation (DCX, neuroD1 BDNF, and trkB). After sertraline feeding we noted a small but significant upregulation of DCX in surgical control animals as compared to water fed controls (Fig. 3D), but not in those previously vagotomised. Expression of the other 3 genes was not altered.

### Sertraline treatment alters diversity of gut microbiota independent of vagotomy

**Figure 4:**
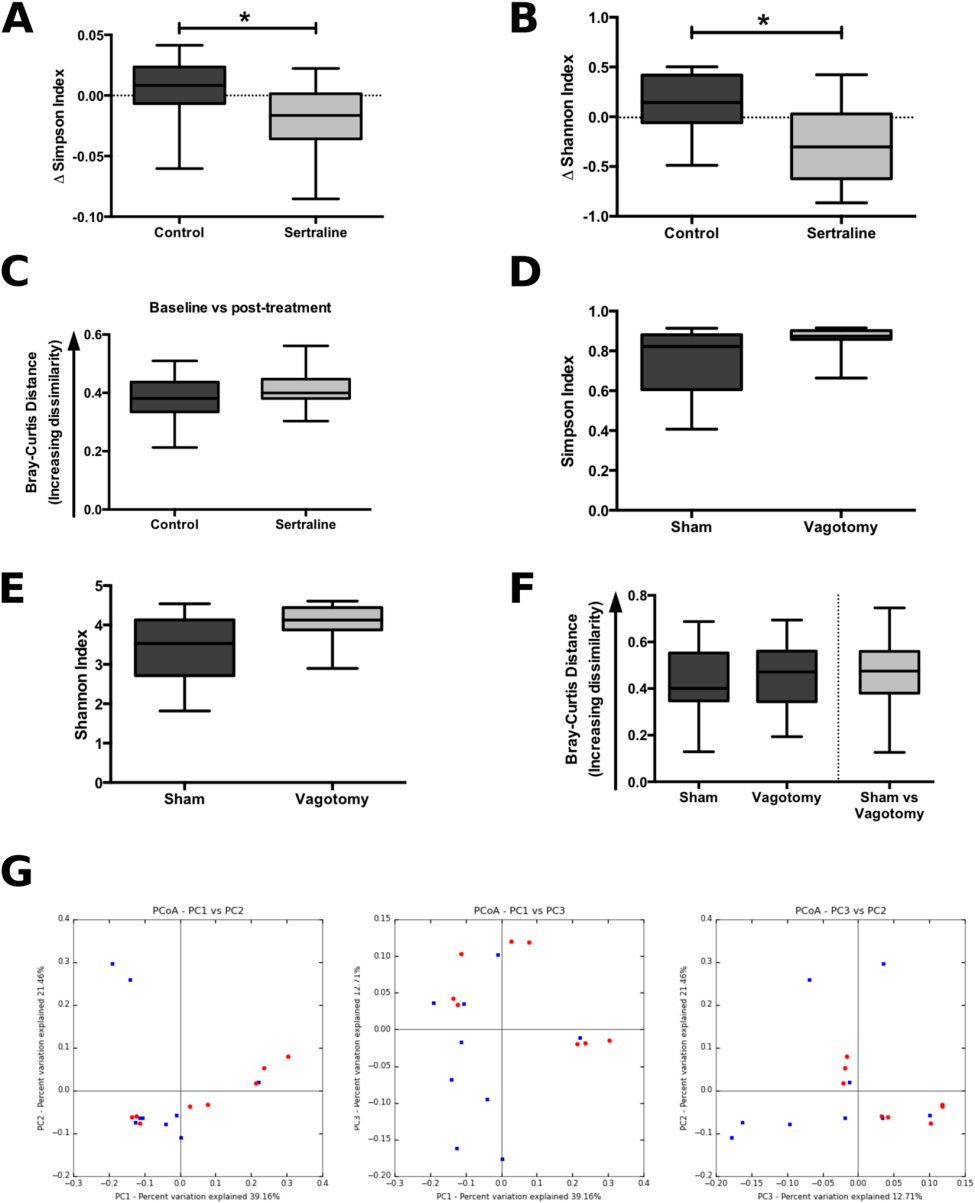
Sertraline, but not vagotomy, induce a decreased microbial richness. (**A**,**B**) Effect of sertraline treatment on the change in alpha-diversity from baseline to post-treatment: (Simpson index, Mann-Whitney U = 31, p = 0.017; Shannon index, Mann-Whitney U = 31, p = 0.017), 4675 reads/sample. (**C**) Bray-Curtis distances from rarefied 16S rRNA data (4675 reads/sample) comparing distances between baseline and post-treatment time-points for control and sertraline-treated mice (Mann-Whitney U = 55, p = 0.343). (**D**,**E**) Effect of vagotomy versus sham surgery on alpha-diversity metrics: (Simpson index, Mann-Whitney *U* = 17, *p* = 0.074; Shannon index, Mann-Whitney *U* = 19, *p* = 0.113), 36040 reads/sample. (**F**) Bray-Curtis distances from rarefied 16S rRNA data (36040 reads/sample) comparing within-group distances of sham surgery and vagotomised groups, and showing distances between sham and vagotomised groups (Mann-Whitney *U* = 457, *p* = 0.529). (**G**) Principal coordinates analysis (PCoA) plots of Jackknifed Bray-Curtis dissimilarity distances from rarefied 16S rRNA data (n = 999 rarefactions, 36040 reads/sample) with PERMANOVA analysis revealing a lack of clustering of samples by group as a result of vagotomy (F = 1.989, *p* = 0.082), (red: sham; blue: vagotomy). *P < 0.05.

Sertraline-treated mice showed a significant decrease in microbial diversity in fecal pellets over the course of treatment as measured by both the Simpson and Shannon indices of diversity (Fig. 4A,B), while within-subject comparison of the change in community structure across the course of treatment revealed that sertraline did not significantly alter the overall microbial profile (Fig. 4C). Vagotomy alone did not significantly alter the alpha-diversity (richness, and evenness) of the gut microbiota compared to sham surgery (Fig. 4D,E). The overall structure of the microbial community was also unchanged as measured by Bray-Curtis dissimilarity (Fig. 4F), and no significant differences were observed in intra-group variability (Fig. 4G). Sertraline treatment and vagotomy both altered the abundance of specific operational taxonomic units (OTUs; Additional File 1: Fig S2 and S3).

**Figure 5:**
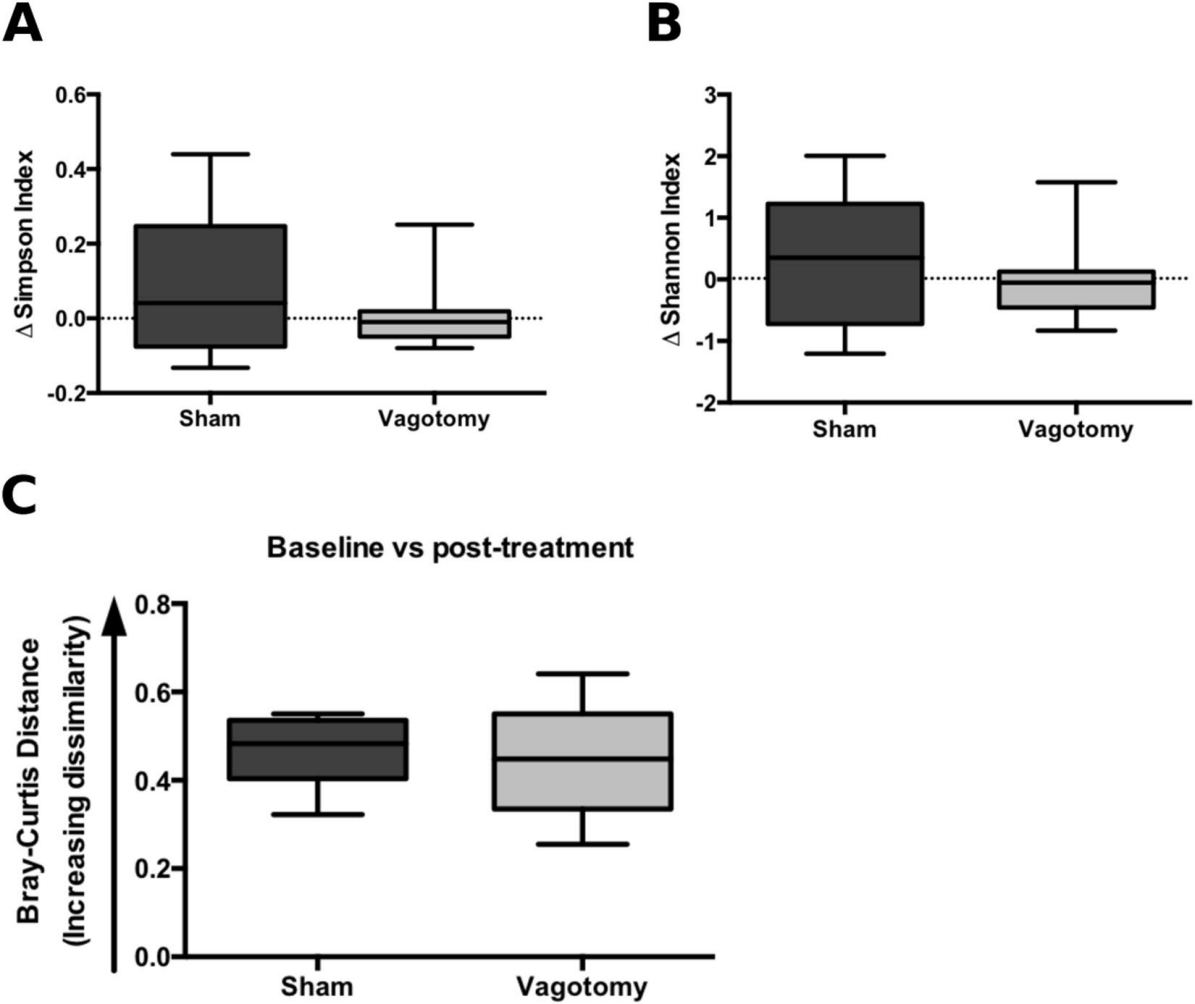
The vagus nerve does not modulate the effect of sertraline on the gut microbiota. (**A**,**B**) Effect of vagotomy versus sham surgery on the change in alpha-diversity from baseline to post-sertraline treatment; Simpson index, Mann-Whitney U = 26, p = 0.366; Shannon index, Mann-Whitney U = 27, p = 0.417, 18499 reads/sample. (**C**) Bray-Curtis distances from rarefied 16S rRNA data (18499 reads/sample) comparing distances between baseline and post-treatment time-points for sham and vagotomised groups, Mann-Whitney U = 28, p = 0.473.

We then examined whether the vagus influenced the effect of sertraline on the microbiome. There was no significant difference between sertraline-treated sham and vagotomised mice with respect to the gut microbiota community diversity (Fig. 5A,B) or overall community profile (Fig. 5C), thus indicating that the intact vagus nerve does not modulate the effect of sertraline on the gut microbiota.

## Discussion

In the current study, we demonstrate that oral treatment with SSRI leads to modulation of vagus nerve activity and that intact vagal signaling between the gut and brain is required to mediate the behavioral effects of these drugs in the TST, a commonly used screen for potential antidepressants.

In order to approach the question of whether SSRI were influencing the activity of the vagus nerve we first examined effects of oral administration of SSRI on the firing frequency of vagal afferent fibres. To achieve this we employed a model system we have used before^24^ of electrical recording (action potentials) of identified vagal afferent fibre single units in mesenteric nerve bundles attached to intact gut jejunal segments, *ex vivo*. We showed that oral feeding of both sertraline and fluoxetine but not the NDRI bupropion, increased the firing frequency of afferent vagal fibres. These vagal effects were entirely reproduced acutely *ex vivo* by luminal presentation of drugs in jejunal gut segments from untreated mice. We note here that while both oral and intraluminal treatment with SSRI increased vagal afferent signalling, this does not explicitly prove that the antidepressive response is entirely dependent on these effects. It remains a possibility that SSRI may exert their antidepressant effects on the brain via some other as yet unknown vagal-dependent mechanism. However, this is unlikely in our opinion in light of previous findings of the antidepressant effects of external vagal stimulation^1^. In further support of our contention are our own findings in the present paper demonstrating the absence of SSRI effect following vagotomy.

In addition to SSRI induced increased vagal firing, here we showed conclusively that the successful afferent vagal neurostimulation by SSRI depends on epithelial signals to the IPANs in the myenteric plexus of the ENS. In this model 2 compartment system^24^, the intact gut is separated from the compartment containing myenteric plexus neurons, which are patch clamped. Only SSRI applied to the epithelium evoked action potentials in the IPANs. Application of SSRI directly on the myenteric neurons failed to produce this effect. Testing of bupropion in this system revealed no evoked action potentials in myenteric neurons with the drug applied to either compartment. We have shown elsewhere that activation of vagal afferents in the gut by luminal bacteria occurs indirectly via a functional nicotinic synapse between IPANs in the myenteric plexus of the ENS and the vagus nerve^10^. We therefore hypothesize that luminal SSRI likely activate vagal afferents in a similar indirect fashion, to eventually signal the brain. However it remains a possibility that the few vagal afferent fibres that are found in the immediate vicinity of the basal aspects of the gut epithelium may be directly affected by the SSRI.

We then demonstrated that oral administration of two SSRI, fluoxetine and sertraline, abolished the increased time spent immobile typically displayed by BALB/c mice^15^, a widely accepted measure of learned helplessness in rodents as assessed by the well-established TST. This therapeutic antidepressant effect was not seen in mice previously subjected to subdiaphragmatic vagotomy. However parenteral injection of an SSRI retained effectiveness in vagotomised animals, suggesting that SSRI could enter the brain and function as anticipated, but that oral SSRI were effectively signalling the brain only via an intact vagus nerve. Accordingly we tested whether integrity of the vagus nerve was essential for the behavioural effect of oral bupropion. Prior vagotomy did not inhibit the behavioural effect of the NDRI.

Oral SSRI have been reported to promote hippocampal neurogenesis and we did record a modest but significant upregulation of DCX but not 3 other gene biomarkers of neurogenesis in the hippocampus. Of note is the considerable disagreement in the literature regarding the possible role of hippocampal neurogenesis in response to SSRI and in depression^41,42^ and recent data have stressed the importance of gut vagal sensory signalling in regulation of hippocampal function^43^. Indeed, a recent evaluation of electrical vagal stimulation for intractable depression has supported its prolonged clinical effectiveness^44^.

There is currently intense interest in the role of the gut microbiota in modulating brain function, particularly in relation to anxiety and depression^45,46^. While sertraline treatment did not alter the overall microbial profile, there was a significant decrease in alpha-diversity over the treatment period that was not observed in controls. Vagotomy did not alter alpha-diversity, nor mediate the effect of sertraline on the microbiome. Intriguingly, intestinal microbiota may modulate the therapeutic function of some psychiatric drugs such as olanzapine^47,48^ and many drugs may exert their actions through effects on microbiota^49^. In fact, a recent publication screening over 1000 marketed drugs against 40 gut bacterial species found that 27% of non-antibiotics inhibited at least one bacterial species’ growth, with psychiatric drugs highlighted as a particularly overrepresented category^50^. Our findings of a significant effect of sertraline on gut microbial diversity is interesting and may indicate that further pursuit of this may be fruitful, but this is beyond the scope of the present research. It remains a possibility that functional effects of SSRI may be modulated by components of the gut microbiome, as has been separately shown for the pharmacologic effects of metformin and cyclophosphamide^51,52^. However we believe this to be unlikely since we show above that SSRI promote vagal stimulation *ex vivo* in washed gut segment recordings within minutes of luminal perfusion.

## Conclusions

Our results lend weight to the possibility that the vagal pathway connecting gut to brain may provide a novel chemotherapeutic opportunity for treatment of some psychiatric disorders. While further study is both necessary and ongoing, we believe that these findings may point towards a newly invigorated approach in the continuing search for new drugs, dietary supplements or bacteria to beneficially modulate these conditions through their effects on vagal afferent communication.

## Supplementary information

Supplementary File

## Data Availability

The data generated and analyzed during this study are available from the corresponding author upon reasonable request.
