# Supplementary material for: Oral selective serotonin reuptake inhibitors activate vagus nerve dependent gut-brain signalling
Source: Sci Rep. 2019 Oct 3;9:14290. doi: 10.1038/s41598-019-50807-8 (PMC6776512; doi:10.1038/s41598-019-50807-8)
Supplement: Supplementary file 1 — Supplementary File [file 41598_2019_50807_MOESM1_ESM.docx]

**Oral selective serotonin reuptake inhibitors activate vagus nerve dependent gut-brain signalling**

Karen-Anne McVey Neufeld*^1,2^, John Bienenstock^1,2^, Aadil Bharwani^1,3^, Kevin Champagne Jorgensen^1^, YuKang Mao^1^, Christine West^1^, Yunpeng Liu^1^, Michael G. Surette^4,5^, Wolfgang Kunze^1,6,7^, Paul Forsythe*^1,4,8^

^1^McMaster Brain-Body Institute at St Joseph’s Healthcare Hamilton, Canada. ^2^Department of Pathology and Molecular Medicine, McMaster University, Hamilton, Canada. ^3^Michael G. DeGroote School of Medicine, McMaster University, Hamilton, Canada. ^4^Department of Medicine, McMaster University, Hamilton, Canada. ^5^Farncombe Family Digestive Health Research Institute, McMaster University, Hamilton, Canada. ^6^Department of Psychiatry and Behavioural Neurosciences, McMaster University, Hamilton, Canada. ^7^Department of Biology, McMaster University, Hamilton, Canada. ^8^Firestone Institute for Respiratory Health, St Joseph’s Healthcare Hamilton, Canada.

**Abbreviated Title:** Serotonin reuptake inhibitors depend on vagus

**Keywords:** vagotomy, serotonin, SSRI, depression, vagus, microbiome

**Corresponding Author:**

Karen-Anne McVey Neufeld

T3330 McMaster Brain-Body Institute, Juravinski Tower

St Joseph’s Healthcare

50 Charlton Ave. E

Hamilton, Ontario, Canada

L8N 4A6

[neufelk@mcmaster.ca](mailto:neufelk@mcmaster.ca)

**Additional File 1**

**Supplementary**

**Supplementary Figure Legends and Supplementary Figures & Tables**

**
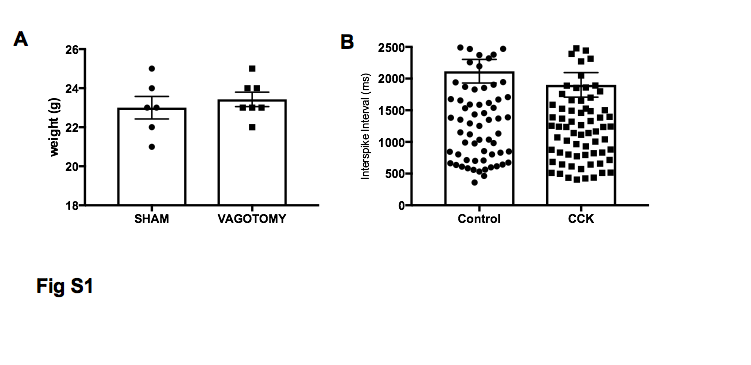
**

**Fig. S1** **Vagotomy does not cause changes in weight in BALB/c mice as compared to sham surgery and persists for at least 4 weeks.** (A) Weight of animals 2 weeks post surgery, presented in grams. Data is means + SEM, n=6/group, unpaired t-test. (B) Absence of increase in nerve activity following CCK indicates no vagal regrowth. Mean interval between single vagal spike firing (ms) for luminal Krebs control and serosal addition of cholecystokinin-8. Mice were vagotomised 4 weeks prior. Data is means + SEM, n=84(6 mice); paired t-test.

|  |  | |  |  | |
| --- | --- | --- | --- | --- | --- |
| **Elevated following vagotomy surgery** | | | **Decreased following vagotomy surgery** | | |
| **OTU 90** | | Lactobacillus | **OTU 101** | | Staphylococcus |
| **300** | | Bifidobacterium | **139, 623** | | Coriobacteriales |
| **92, 124, 251, 316** | | Lachnospiraceae | **123** | | Lachnospiraceae |
| **160** | | Clostridiales | **35** | | Akkermansia |
|  | |  | **327** | | Adlercreutzia |

**Fig. S2** **Differentially represented OTUs following sertraline treatment.**

| **OTU** | **Elevated following sertraline treatment** | **OTU** | **Decreased following sertraline treatment** |
| --- | --- | --- | --- |
| **24, 105, 121** | Ruminococcaceae | **34, 177** | Clostridiales |
| **38** | Bacteroidales | **42** | Catabacteriaceae |
| **65** | Bacteroides | **62, 66, 179** | Lachnospiraceae |
| **99, 165, 290** | Lachnospiraceae | **136** | Adlercreutzia |
| **224, 228** | Clostridiales | **196** | Erysipelotrichaceae |
| **226** | Alphaproteobacteria | **330** | Alphaproteobacteria |
| **290** |  | **418** | Ruminococcaceae |
|  |  |  |  |

**Fig. S3 Differentially represented OTUs following** **vagotomy.**
